# Practical Remarks on the Diseases of the Skin, on the External Signs of Disorder, and on the Constitutional Peculiarities during Infancy and Childhood

**Published:** 1839-01-01

**Authors:** 


### PRATICAL REMARKS ON THE DISEASES OF THE SKIN, ON THE EXTERNAL SIGNS OF DISORDER, AND ON THE CONSTITUTIONAL PECULIARITIES DURING INFANCY AND CHILDHOOD. By *Walter C. Dendy*, Surgeon to the Royal Infirmary for Children, &c. 8vo. pp. 153. 1838.

MR. DENDY, a very zealous and intelligent member of our profession, offers in his Preface, the following reasons for the publication of the present work.

"In medical literature, a profusion of opinions or arguments detracts from this facility, by too often leaving the adoption of practical modes to the reluctant decision of the reader—the very profusion thus limiting the sphere of utility. I have therefore endeavoured, here, to improve the remarks in my former work on these subjects; to present a book, not of argument, but of practical precepts, founded on long observation; a volume for reference, rather than of elaborate study."

The cutaneous pathology of the treatise essentially regards the diseases, as they occur in childhood. Mr. Dendy has deservedly enjoyed much experience in the complaints incidental to this period of life. We shall therefore select such passages as may offer, on several points, its results.

1. *On the External Signs of Internal Disorder.*—We find some observations on this head, some of which are neither uninteresting, nor uninstructive. And first of the expression of *feature.* Mr. Dendy observes:—

In the progress of hooping-cough, the eye should be attentively regarded. While the disease is confined to the organs of breathing, the eye will be little influenced, except in the redness subsequent to a paroxysm of cough; but we may almost decide if the brain be about to participate, according to the *fixed* contraction or dilatation of the pupil.

Strabismus suddenly taking place, in connexion with other symptoms, is an indication of danger. Its *gradual* and *unattended* progress may arise from mere irritation from worms or other slight disorders of the bowels, or from rays of light constantly falling on the eye from one direction.

*Contraction* of the pupil to a minute point, with the eye half closed, a red streaked condition of the conjunctiva, with frowning or knitting of the brows, with spasm of the muscles of the globe; this combination unequivocally marks that condition which will rapidly become inflammation of the membranes of the brain, the first stage of *acute hydrocephalus.*

Mr. D. believes that the spasm of the globe indicates the presence of arachnitis, and that about the base of the brain,

When the eyeball is fixed and drawn up under the lid, the pupil widely dilating and contracting, the eye being bright and glassy, we may often anticipate, if not averted, convulsion or infantile epilepsy. Inequality of action of the pupil on exposure to the light is dangerous.

Any peculiar movement, continues, Mr. D., about the nose and lips indicates disorder about the chest and abdomen.

If there be impediment to the transmission of air through the lungs, the nose will be drawn in during respiration; the mouth being unusually open, and the lips often puckered, and of a livid hue.

When the nose and upper lip are tumefied, there is irritation of the bowels, probably arising from the presence of worms.

If the inside of the nose be dry, and the lips pale and cracked, our attention should be immediately paid to the condition of the head. And when we see frequent spasm of the lower jaw, we may then anticipate that the base of the brain is threatened.

The movements of the limbs furnish indications of disease. There is in pleuritis, for instance, observes Mr. Dendy, often a doubling up of the body to relieve tension, and the legs are *forcibly* drawn up towards the belly, when inflammation of the serous or mucous membranes of the lower bowels is present. If *one* limb, however, is unusually quiet, or is moved by sudden jerks, that is probably the *seat* of pain.

"As early as the fifth or sixth day, infants will sometimes be attacked by spasms in the muscles of the face, lower jaw, or neck, and in severe cases we have seen the jaw completely fixed. This is often, I believe, the effect of retention of meconium, or extending ulceration of the umbilical cord.

Convulsion will arise also from painful dentition, when it is often attended by swelling of the head and feet, from acidity in the bowels, and the effort to throw out eruptive diseases on the skin. The legs are then often rigidly extended during sleep, these effects subsiding when the eruption has appeared. A contraction of the thumbs, fingers, and toes (carpo-pædal spasm) is often the premonitory sign of more severe convulsion, and opisthotonos, the attendant of that form of cerebral irritation marked by *crowing*." 13.

In older children, he continues, an uplifted step, a staggering gait, and a rocking of the legs, often indicate that species of hydrocephalus which occurs without *acute* disorder; and if they waver much from side to side, the medulla oblongata is usually affected.

After some remarks on the indications offered by the voice, by altered states of respiration, (in which he includes the "laryngismus stridulus,") Mr. D. sums up with the following brief observation :—

When a cough assumes the *spasmodic* character, it is usually a mark of cerebral affection, as cough with *mucous expectoration* is of pulmonary or bronchial, and a dry and *irritative* cough of gastric disorder.

To proceed to the Diseases of the Skin, Mr. Dendy sets out with two classifications—one, of these diseases according to their character—the pustular, the scaly, and so on; the other, of the diseases according to their causes. Could such an arrangement be rendered unobjectionable, it must be a highly useful one, for it would present at one view both a philosophical classification of diseases, and one which would point directly to remedial means. We present Mr. Dendy's, and shall offer a remark or two upon it.

### ARRANGEMENT ACCORDING TO CAUSES.

| | | |
|---|---|---|
| Diseases symptomatic chiefly of disorder of the alimentary canal, marked by *increased* cutaneous action, often by subacute or chronic inflammation. | Occurring during dentition or suckling . . | Strophulus. |
| | | Lichen. |
| | | Prurigo. |
| | | Crusta lactea. |
| | | Impetigo. |
| | Dependent chiefly on gastro-enteric irritation. | Roseola. |
| | | Erythema. |
| | | Eczema. |
| | | Urticaria. |
| | | Erysipelas. |
| | | Phlegmon. |
| | | Herpes. |
| | | Lepra. |
| | | Psoriasis. |
| | | Crinones. |
| | | Verruca follicularis. |
| | | Acne. |
| | | Sycosis. |
| | | Porrigo. |
| Diseases indicative of debility, marked by *languid* cutaneous action, often the sequelæ of acute disorder. | Original debility of the system . . . . . . | Pityriasis. |
| | | Icthyosis. |
| | | Elephantiasis, |
| | | Alopecia. |
| | | Shrivelled skin. |
| | | Ephidrosis. |
| | Derangement of the chylopoietic function. | Apthæ. |
| | | Miliaria. |
| | | Ecthyma. |
| | | Rupia. |
| | | Pemphigus. |
| | | Anthracion. |
| | | Anthrax. |
| | | Purpura. |
| | | Nomé. |
| | | Struma. |
| | | Onychia maligna. |
| | | Chloasma. |
| Diseases consequent to specific infection. | Febrile . . . . . | Rubeola. |
| | | Scarlatina. |
| | | Varicella. |
| | | Variola. |
| | Non-febrile . . . | Vaccinia. |
| | | Scabies. |
| | | Syphilides. |

| | | |
|---|---|---|
| Diseases consequent to external and common irritation. | . . . . . . . . | Encausis. Vesication from external irritants. Pernio. Paronychia. Pterygion. Verruca. Clavus. Intertrigo. Rhaghades. Condyloma. |
| Maculæ . . . | | Nævus vascularis. Lentigo. Tinge of Argenti Nitras." xii. |

The two great classes are the first and second. We confess that the allocation of particular affections appears to us, in many instances, extremely arbitrary. Thus, in the same category, we find roseola and sycosis, erythema and phlegmon, acne and lepra. All these are said to depend chiefly on gastro-enteric irritation. But why should lepra be referred to such irritation rather than strophulus, or impetigo? And why should pityriasis be attributed to original debility of the system, rather than psoriasis? This arrangement of diseases of the skin refers all to the interior. Peculiarities of the structure of the skin go for nothing. This appears to us as fatal an error as referring all to the skin itself, and nothing to the system. The skin is a very extensive, highly organized, and powerful secreting organ. It is exposed particularly to the causes of disease. Why should not disease originate in *it* sponte suâ. A priori, it is likely that it would do so, and experience, we think, corroborates this view. The mucous membranes, the glands, are, after all, but internal integuments. If, in a large number of cases, cutaneous complaints have their source in derangements of the interior, in a large number, also, they depend on a vicious conformation or disturbance of the dermoid tissue itself. A dry skin is the fruitful cause of eruptions. That dryness is often combined with general or with internal derangements—but in many cases, there is no evidence in favour of its being so.

But arrangements of cutaneous diseases are notoriously beset with difficulties. It will be long ere any perfectly satisfactory one will be produced. The study of the affections themselves is the main thing.

We have looked over the work and we must say that the diseases are sketched with distinctness, while the medical directions are precise. We shall extract those for the treatment of porrigo scutulata, pustular ringworm.

Premising, of course, proper constitutional treatment, our author advises that—

The local treatment should be commenced with warm water washes, and roll poultices, for three or four days; the loose hairs should then be detached by forceps; and, if there be no inflammatory condition, recourse should then be had to more stimulant treatment; but patience and perseverance are much required. I append recipes of several forms; premising, that it is essential to wash off gently with soft flannel the previous applications, before employing the fresh ones.

> Acid. nitric. ʒss. ad ʒj. Aq. dist. ℥i. ad ℥iij.

> Cupri sulphat. pulv. rubbed in lightly.

These two applications should, if they produce pain, be washed off ten minutes after their application.

> Liq. plumb. subac.—Acid. acet.—T. opii, ā ʒiij. Mist. camphoræ, lb. ss.

> U. Hydr. nit. m. ℥j. Camphor. ʒss. Sulphur. ʒj.

> Potassæ sulphuret. ʒss. Aq. dist. ℥j.

> Chloruret sodæ solut. in aq. distill.

> Sodæ alicant. ʒiij. Potass. sulphuret. ʒiij. Aq. ℥iij.

> Acid. acet. (tepid) o. n. Ungt. hydr. n. m. mane.

If the crusts are very dry, liq. potassæ should be applied, and subsequently a warm poultice; after this, the infus. gallæ, to restore healthy action. In cases of several months' or years' standing, I apply a blister for 24 hours, for the purpose of changing completely the cutaneous tissue; it need not be kept open above a day or two, if it has effectually penetrated the deeper textures." 68.

Mr. Dendy does not mention an application which we have found beneficial, particularly in the porrigo decalvans—the ointment of the ioduret of sulphur. It is made of one part of "ioduret of sulphur," and eighteen to twenty of lard. It should be rubbed on the part night and morning very gently.

We recommend Mr. Dendy's as a practical book.

